# Luxation of Both Shoulders

**Published:** 1877-01

**Authors:** 


Luxation of Both Shoulders (Cossy: *Bulletin de la Societe Anatom. de Paris*, 1875).—A patient suffering with phlegmonous abscess of the leg applied for treatment, and upon examining him it was found that both shoulders had been dislocated, the right for five years, the left for three weeks. The patient died suddenly, and at the *post mortem* examination the parts surrounding the joint of the left side were in good condition; there was a rupture of the capsular ligament, which was not large enough to permit the head of the humerus to pass through it, and the synovial membrane was reddened, especially around the head of the humerus and along the long tendon of the biceps. On the right side no peri-articular changes were found, nor any tear in the capsular ligament. Upon opening the cavity of the joint, the articular surface was found to have become smaller, and was covered with whitish granulations and separated from it by a ridge. There was seen upon the inner surface of the scapula a somewhat wider new articulating surface. The head of the humerus had changed its shape, being convex on its inner side, which corresponded with the new articular cavity, while the posterior part, which corresponded with the glenoid cavity, was concave.

